# Antinociceptive Effect of Tetrandrine on LPS-Induced Hyperalgesia via the Inhibition of IKKβ Phosphorylation and the COX-2/PGE_2_ Pathway in Mice

**DOI:** 10.1371/journal.pone.0094586

**Published:** 2014-04-10

**Authors:** Hengguang Zhao, Fuling Luo, Hongzhong Li, Li Zhang, Yongfen Yi, Jingyuan Wan

**Affiliations:** 1 Department of Dermatology, the First Affiliated Hospital of Chongqing Medical University, Chongqing, China; 2 Department of Pharmacy, the First Affiliated Hospital of Chongqing Medical University, Chongqing, China; 3 Molecular oncology and epigenetics laboratory, the First Affiliated Hospital of Chongqing Medical University, Chongqing, China; 4 Department of Pathophysiology, Chongqing Medical University, Chongqing, China; 5 Department of Pathology, Molecular Medicine and Tumor Center, Chongqing Medical University, Chongqing, China; 6 Chongqing Key Laboratory of Biochemistry and Molecular Pharmacology, Chongqing Medical University, Chongqing, China; Chang Gung University, Taiwan

## Abstract

Tetrandrine (TET) is a bisbenzylisoquinoline alkaloid that is isolated from the *Stephania Tetrandra*. It is known to possess anti-inflammatory and immunomodulatory effects. We have shown that TET can effectively suppress the production of bacterial lipopolysaccharide (LPS)-induced inflammatory mediators, including cyclooxygenases (COXs), in macrophages. However, whether TET has an antinociceptive effect on LPS-induced hyperalgesia is unknown. In the present study, we investigated the potential antinociceptive effects of TET and the mechanisms by which it elicits its effects on LPS-induced hyperalgesia. LPS effectively evoked hyperalgesia and induced the production of PGE_2_ in the sera, brain tissues, and cultured astroglia. TET pretreatment attenuated all of these effects. LPS also activated inhibitor of κB (IκB) kinase β (IKKβ) and its downstream components in the IκB/nuclear factor (NF)-κB signaling pathway, including COX-2; the increase in expression levels of these components was significantly abolished by TET. Furthermore, in primary astroglia, knockdown of IKKβ, but not IKKα, reversed the effects of TET on the LPS-induced increase in IκB phosphorylation, P65 phosphorylation, and COX-2. Our results suggest that TET can effectively exert antinociceptive effects on LPS-induced hyperalgesia in mice by inhibiting IKKβ phosphorylation, which leads to the reduction in the production of important pain mediators, such as PGE_2_ and COX-2, via the IKKβ/IκB/NF-κB pathway.

## Introduction

Inflammatory mediators, such as prostaglandins (PGs), PG synthases, and cyclooxygenases (COXs), can cause abnormal neuronal activity, which leads to pain hypersensitivity[1]. Over the past decade, many studies have focused on the roles of these mediators in the regulation of hypersensitivity that is induced by environmental stimuli and pro-inflammatory factors, such as bacterial lipopolysaccharide (LPS)[2].

In the central nervous system (CNS), treatment of astrocytes and microglia with low concentrations of LPS can produce PGE_2_ via Toll-like receptor 4-dependent manners[3], [4]. PGE_2_ can directly trigger pain-sensitive neurons to induce nociception[5], [6]. At the same time, PGE_2_ receptors that are located in peripheral tissues can scatter to the end of nociceptive nerve endings, thus sensitizing the CNS to the existence of nociceptive stimulation[7]. The COXs are rate-limiting enzymes that catalyze the synthesis of PGs. There are two distinct isoforms: COX-1 and COX-2. Although the existence of COX-3 has been reported, its roles and effects in humans are still unclear[8]–[9]. COX-1 is constitutively expressed to regulate normal physiological conditions, whereas COX-2 is initiated in response to inflammatory signals, such as cytokines and LPS. Moreover, in inflammatory pain conditions, COX-2 itself can act as a nociceptive stimulator to directly cause pain. COX-2 is regulated by nuclear factor (NF)-κB, which is a well-known transcription factor that is involved in inflammation or injury. Recent reports revealed that NF-κB is also implicated in hyperalgesia[10]–[11], which is regulated by a series of adaptors. Under normal conditions, NF-κB is inactive, and it is bound to inhibitor κB (IκB) via its subunits, P65 and P50, in the cytoplasm. Upon IκB kinase (IKK) activation, IκB is phosphorylated, thus resulting in its ubiquitination and subsequent degradation by the 26S proteasome. NF-κB then translocates into the nucleus to regulate the transcription of genes that code for inflammatory cytokines and nociceptive substances [12], [13].

**Figure 1:**
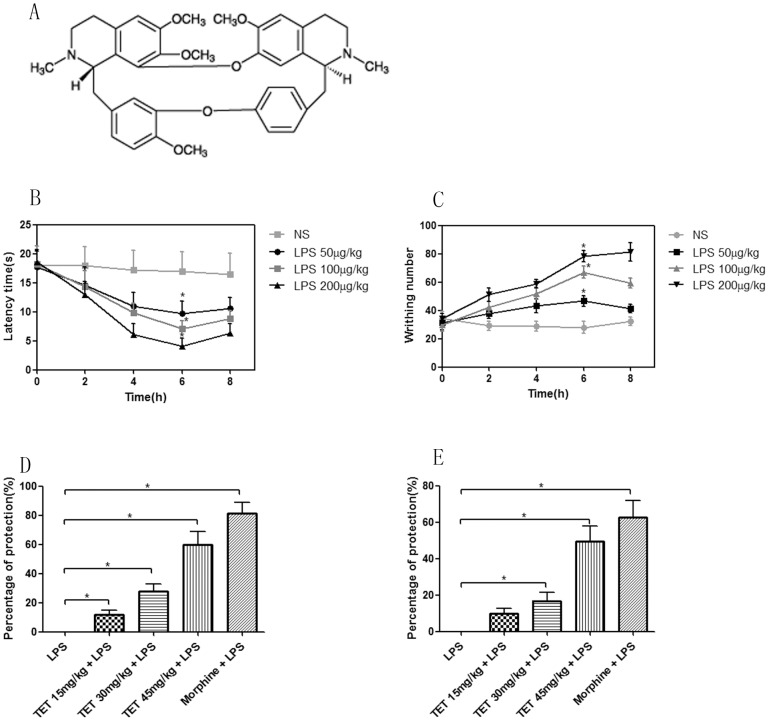
Induction of hyperalgesia by LPS in BALB/C mice. (A) Molecular structure of TET. (B) Latency time of hind-paw licking in the hot-plate test in mice that were treated with LPS at different concentrations and time points. (C) Writhing counts obtained from the acetic acid-induced abdominal constriction test in mice that were treated with LPS at different concentrations and time points. (D and E) Percentages of protection by TET (15, 30, 45 mg/kg) at 6 h after LPS (100 μg/kg) stimulus, as indicated by the hot-plate test (D) or acetic acid-induced abdominal constriction test (E). Indomethacin (5 mg/kg) and morphine (10 mg/kg) were applied as the positive controls. Values are shown as M±SD. *, *P*<0.05.

Tetrandrine (TET) is an important bisbenzylisoquinoline alkaloid that is isolated from *Stephania Tetrandra* (Fig. 1A). It is traditionally used in China and Korea to treat patients with arthritis. Previous studies have shown that it possesses anti-arrhythmic[14], anti-hypertensive[15], cardio-protective[16], anti-tumorigenic[17], and anti-inflammatory effects[18]. We have demonstrated that TET exhibits anti-inflammatory and hepato-protective effects in mice[19], [20], and it inhibits IL-6 and TNF-α production in macrophages. However, whether it is involved in the inflammatory processes of nociception is unknown. In this study, we tested the role of TET on LPS-induced hyperalgesia in mice and investigated the potential mechanisms by which TET elicits its effects.

## Materials and Methods

### 2.1 Animals

BALB/C mice (6–8 weeks old, 20–22 g) were obtained from the Laboratory Animal Center of Chongqing Medical University (Chongqing, China). All mice received humane care, and all studies were performed with approval from the Animal Care and Use Committee of Chongqing Medical University (approval #SCXK20070001). The mice were maintained in a SPF-grade facility under controlled conditions (22°C, 55% humidity, and 12 h day/night rhythm) and fed standard laboratory chow. After each experiment, mice were sacrificed under anesthesia with isoflurane and decapitated to ameliorate any suffering.

### 2.2 Materials and Drug Preparations

TET (C_38_H_42_O_6_N_2_, molecular weight: 622.8 g/mol) was purchased from the National Institute for the Control of Pharmaceutical and Biological Products (Beijing, China), and its purity was determined by HPLC, as previously described[19], [20]. LPS (Escherichia coli, 0111:B4), morphine hydrochloride, and indomethacin were purchased from Sigma (MO, USA). All drugs were freshly prepared on the day of experiments. TET powder was dissolved in 0.01 M hydrochloric acid, and the pH was adjusted to 5.5 with 0.01 M NaOH. The LPS stock solution was reconstituted to a final concentration of 10 μg/ml. Indomethacin solutions for intraperitoneal injections were prepared fresh in 0.01 M sodium carbonate, pH 7.2, at a final concentration of 0.2 mg/ml.

### 2.3 Induction of hyperalgesia in mice

To induce hyperalgesia, mice were randomly divided into the control group (0.1 ml/10 g pyrogen-free sterile saline, intraperitoneal injections [i.p.]), LPS-stimulated group (100 μg/kg, i.p.), low TET+LPS-treated group (15 mg/kg TET, 100 μg/kg LPS, i.p.), moderate TET+LPS-treated group (30 mg/kg TET, 100 μg/kg LPS, i.p.), high TET+LPS-treated group (45 mg/kg TET, 100 μg/kg LPS, i.p.), indomethacin-treated group (5 mg/kg, 100 μg/kg LPS, i.p.), and morphine-treated group (10 mg/kg, 100 μg/kg LPS, i.p.). TET, indomethacin, or morphine was administered 30 min prior to LPS injections. Each group was repeated at least by five individuals.

The hot-plate and acetic acid-induced abdominal constriction (writhing) tests were performed to measure the hyperalgesic responses to LPS in the presence or absence of TET, indomethacin, or morphine. The hot plates (RB200, Chengdu TME, China) were maintained at 55±1°C, and induction time was determined by measuring the latency of paw licking every 2 h until 8 h after treatment. Due to its long-term effects, acetic acid was administered only once for each individual mouse at different time points (0, 2, 4, 6, and 8 h) and concentrations. The acetic acid-saline solution (0.1 ml/10 g of 0.7% acetic acid-saline) was intraperitoneally injected, and the frequency of abdominal constrictions was counted for 20 min. Writhe was defined as the contraction of abdominal muscles, which were accompanied by the extension of forelimbs and elongation of the body.

### 2.4 Treatment and culture of astroglia

To prepare mouse cerebral astrocytes, cerebral cortices from P1 neonatal BALB/C mice were mechanically dissociated in astrocyte culture medium (Dulbecco's Modified Eagle Medium [DMEM] with 10% fetal bovine serum [FBS] and antibiotics). After filtering through a 70 μm cell strainer, the cells were seeded in cell culture flasks. To obtain astroglia, confluent cultures were shaken at 250 rpm overnight at 37°C. The purity of astrocytes was checked by immunostaining for GFAP (Abcam, MA, USA), and the threshold was set at >95%[21], [22]. When the primary cells reached 80–90% confluency, they were digested by 0.25% trypsin and plated in 12-well tissue-culture plates at a density of 1.0–1.5×10^5^ cells/well. When the cells in the 2^nd^ passage were close to confluence, the culture medium was replaced with FBS-free DMEM. Cell treatments were performed according to following groups: control group (only the FBS-free DMEM); LPS-stimulated group (1 μg/ml); low, moderate, and high TET-treated groups (1 μg/ml LPS with TET [1×10^−8^ mol/l, 1×10^−7^ mol/l, and 1×10^−6^ mol/l, respectively]). Treatments lasted for 6 hours, after which the cells and supernatants were harvested for various experiments.

### 2.5 Measurement of cell viability

The TET toxicity of cultured cells was determined using the MTT (3-(4,5-Dimethylthiazol-2-yl)-2,5-diphenyltetrazolium bromide) assay, according to the manufacturer's instructions. In brief, cells were seeded in 96-well plates and treated with increasing concentrations of TET (1×10^−8^ mol/l–1×10^−4^ mol/l) for 15 min. MTT (terminal concentration: 0.5 g/L) was added into each well and incubated for 4 h. The optical density (OD) was measured at 570 nm by microplate UVspectrometer (SpectraMax 384 Plus).

### 2.6 Enzyme Immunoassay (EIA)

PGE_2_ levels in purified plasma, brain homogenates, and cell culture supernatants were evaluated using a commercial EIA kit (Cayman, Michigan, USA), according to the manufacturer's protocol. Samples were added to a plate that was pre-coated with goat anti-mouse IgG antibodies. PGE_2_ monoclonal antibodies were then added to each well, and the plates were incubated for 18 h at 4°C. Afterwards, Ellman's Reagent substrate was added to each well. The optical density of each sample was read at 412 nm. The standard curve was plotted, and the final concentrations of PGE_2_ in the samples were calculated using the equations that were obtained from the curve.

### 2.7 Western blotting

Brain tissues and cultured cells were homogenized in protein extract solution. Protein concentrations were determined using the BCA protein assay kit (Thermo, USA). Samples (40 μg) were loaded onto a 12% polyacrylamide-sodium dodecyl sulfate (SDS) gel and then transferred to a nitrocellulose membrane. The membrane was blocked with 5% (w/v) fat-free milk in Tris-buffered saline (TBS) containing 0.05% tween-20, followed by overnight incubations at 4°C with the primary antibody (1∶1000). Afterwards, the membranes were treated with horseradish peroxidase-conjugated secondary antibody (1∶5000, Abcam, USA) and visualized using an ECL chemiluminescence system with short exposure to X-ray films (Kodak, USA). The following primary antibodies were used: COX-1, COX-2, P65, phosphorylated P65 (pP65), IκBα, phosphorylated IκBα (pIκBα), IKKα, phosphorylated IKKα (pIKKα), IKKβ, phosphorylated IKKβ (pIKKβ), GAPDH, and tubulin (Abcam, USA).

### 2.8 Small-interfering RNA (siRNA) transfection

The SignalSilence siRNAs of IKKα, IKKβ, and control siRNA (unconjugated) were obtained from Santa Cruz Biotechnology (CA, USA). Cells were plated onto a 6-well plate at a density of 1.6×10^5^ cells/well. Once they were 60–80% confluent, cells were washed with PBS, and the pre-mixed siRNA transfection solution (including siRNA duplex solution and the dilution reagent) was added directly to the culture medium. Cells were then incubated for 24 h, and the culture medium was changed for another 24 h. The reactions were stopped, and lysis buffer was added to extract proteins from the cells for further experiments.

### 2.9 Quantitative Real-Time Polymerase Chain Reaction (qRT-PCR)

Total RNA was isolated from brain tissues and cultured cells using Trizol reagent, (Invitrogen, USA) according to the manufacturer's guidelines, followed by further purification using the RNeasy Mini Kits (Qiagen, USA). Purified RNA was reverse-transcribed into cDNA with random hexamers by the SuperScriptTM First-Strand Synthesis System kit (Qiagen, USA) and analyzed by real-time RT-PCR with the QuantiTect SYBR Green PCR Kits (Qiagen, USA), using a MJ DNA Engine Opticon2 qPCR System (MJ, USA). The following oligonucleotide primers were used: COX-1 forward: 5′-TGC CCT CTG TAC CCA AAG AC-3′, reverse: 5′-GGA CCC ATC TTT CCA GAG GT-3′; COX-2 forward: 5′-CGG AGA GAG TTC ATC CCT GA-3′; reverse: 5′-ATC CTT GAA AAG GCG CAG T-3′; GAPDH (control) forward: 5′-CTC ATG ACC ACA GTC CAT GC-3′, reverse: 5′-CAC ATT GGG GGT AGG AAC AC-3′.

### 2.10 Statistical Analysis

All data were expressed as mean±standard deviation (M±SD) from at least four independent experiments. Results were analyzed by Student's *t* test or analysis of variance (ANOVA). *P*≤0.05 was considered to be statistically significant.

## Results

### 3.1 LPS induced hyperalgesia in mice via time- and dose-dependent manners

The administration of LPS (50, 100, or 200 μg/kg, i.p.) evoked dose-dependent hyperalgesia, as evaluated by the hot-plate and acetic acid-induced abdominal constriction tests (Fig 1). In the hot-plate test, hyperalgesia was assessed by the action of hind-paw licking. A decrease in the latency time of paw licking was observed at 2 h after LPS treatment, and maximal reduction was observed at 6 h (from 17.0 to 9.7, 7.1 and 4.1 seconds at LPS of 50, 100, 200 μg/kg, respectively). This was maintained until 8 h after LPS treatment (Fig 1B). Acetic acid-induced abdominal constriction numbers increased with LPS treatment, and the analogous initial and peak time points were determined (Fig 1C). Based on these results, we chose the 6-h time point for further experiments. In addition, mice that were exposed to the highest concentration of LPS (200 μg/kg) exhibited obvious hyperalgesia. However, this was accompanied by adverse reactions, including cachexia, diarrhea, and sustained tumbling, all of which may potentially influence the evaluation of the algesic effect. Alternatively, a lower dose of LPS (100 μg/kg) induced obvious hyperalgesia without the adverse effects. Therefore, this concentration of LPS was used for further experiments.

### 3.2 Analgesic effects of TET on LPS-induced hyperalgesia in mice

Different doses of TET were administered to mice 30 min prior to LPS treatment. LPS-induced hyperalgesia was significantly repressed, as indicated by the elongated threshold time in hot-plate tests and the decrease in the number of writhing in acetic acid-induced abdominal constriction tests. The percentages of protection at 6 h by TET concentrations of 15, 30, and 45 mg/kg were 11.7%, 27.8%, and 59.6%, respectively, in the hot-plate test (Fig. 1D) and 9.7%, 16.8%, and 49.6%, respectively, in the acid-induced abdominal constriction test (Fig. 1E). Indomethacin (5 mg/kg) and morphine (10 mg/kg) were used as the positive controls, due to their known antinociceptive effects[23], [24]. The percentages of protection by morphine and indomethacin were 81.2% (hot-plate test) and 62.7% (acid-induced abdominal constriction test), respectively. These results indicate that TET may possess both peripheral and central antinociceptive properties on LPS-induced hyperalgesia in mice.

### 3.3 TET repressed PGE_2_ production in LPS-induced hyperalgesia in mice and cultured astroglia

**Figure 2:**
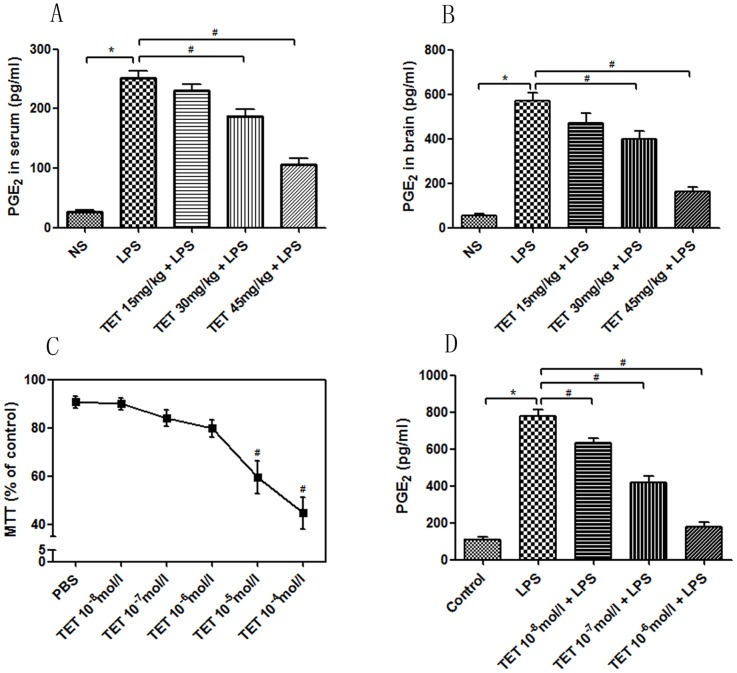
Repressive effects of TET on PGE_2_ levels in sera, brain tissues, and cultured astroglia. (A, B) PGE_2_ levels in the sera and brain tissues, respectively, were significantly increased after LPS stimulation and markedly suppressed by TET. (C) MTT analysis of astroglia viability *in vitro* in the presence of different concentrations of TET. (D) PGE_2_ levels in LPS-treated astroglia were suppressed by TET in a dose-dependent manner. Values are shown as M±SD. *, *P*<0.05.

To explore the antinociceptive mechanism of TET, PGE_2_ production was measured by EIA in the sera and brain tissues of LPS-induced hyperalgesic mice and in the supernatants of cultured astroglia. *In vivo*, LPS significantly increased PGE_2_ levels, which were markedly suppressed by TET pretreatment in dose-dependent manners (Fig. 2A, 2B). To exclude the pathophysiological conditions *in vivo* that may potentially affect the intrinsic reactions, we cultured astroglia cells to verify the mechanism *in vitro*. The toxicity of TET on astroglia was evaluated by the MTT assay, and we confirmed that TET concentrations from 1×10^−8^ mol/l to 1×10^−6^ mol/l did not significantly repress cell viability (Fig. 2C). Therefore, these concentrations of TET were used in further experiments. Treatment of astroglia with 1 μg/ml LPS significantly increased PGE_2_ levels. Similarly, TET co-treatment attenuated PGE_2_ levels in a concentration-dependent manner. The repressive peak was at the TET concentration of 1×10^−6^ mol/l (Fig. 2D). These results suggest that the antinociceptive effect of TET on LPS-induced hyperalgesia in mice may be partially mediated through downregulation of the PGE_2_ signaling pathway.

### 3.4 TET suppressed COX-2, but not COX-1, levels

**Figure 3:**
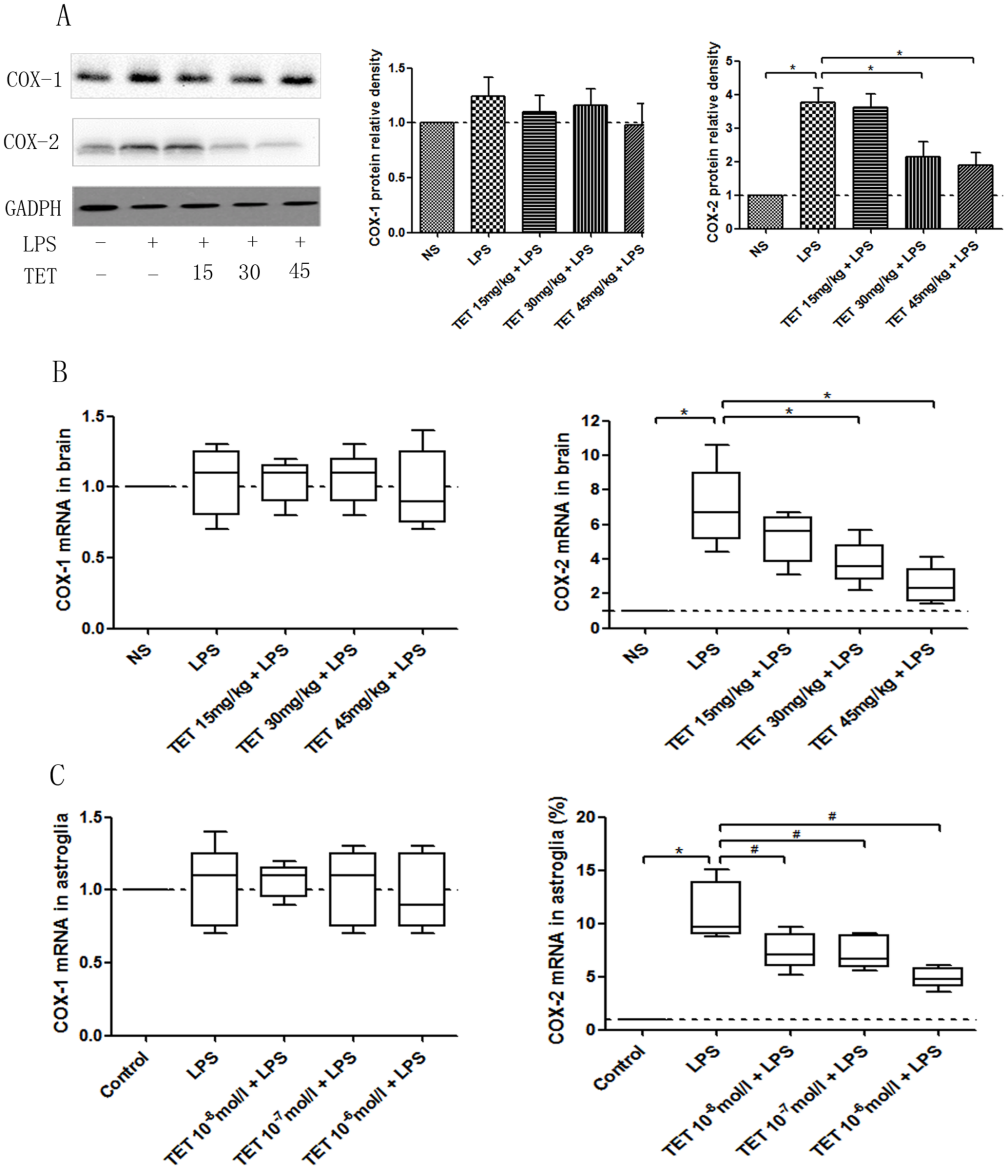
Repressive effects of TET on the expression of COX-1 and COX-2 in LPS-induced hyperalgesia. (A) Western blotting of COX-1 and COX-2 in brain tissues and the quantified comparison of relative densities are shown. (B) COX-1 and COX-2 mRNA expression were tested by qRT-PCR in brain tissues. (C) COX-1 and COX-2 mRNA expression were tested by qRT-PCR in cultured astroglia. Values are shown as M±SD, and normalized to the NS groups. *, *P*<0.05.

Following its release from membrane phospholipids by cytosolic or secretory phospholipases, arachidonic acid is converted to PGE_2_ by COX-1 and COX-2[25]. We then investigated the expression of COX-1 and COX-2 at the mRNA and protein levels. As shown in Figure 3A, brain tissues from LPS-stimulated mice exhibit four-fold increases in COX-2 protein levels, and TET pretreatment decreases these levels of COX-2. No changes in COX-1 were observed in the presence or absence of LPS or TET. This indicates that TET can selectively suppress COX-2 expression. Similar trends in the mRNA levels of COX-1 and COX-2 were observed (Fig. 3B and 3C). Thus, the mechanisms of action in vivo and in vitro appear to be the similar processes.

### 3.5 TET decreased COX-2 expression through IKKβ, which further inhibited the NF-κB pathway

**Figure 4:**
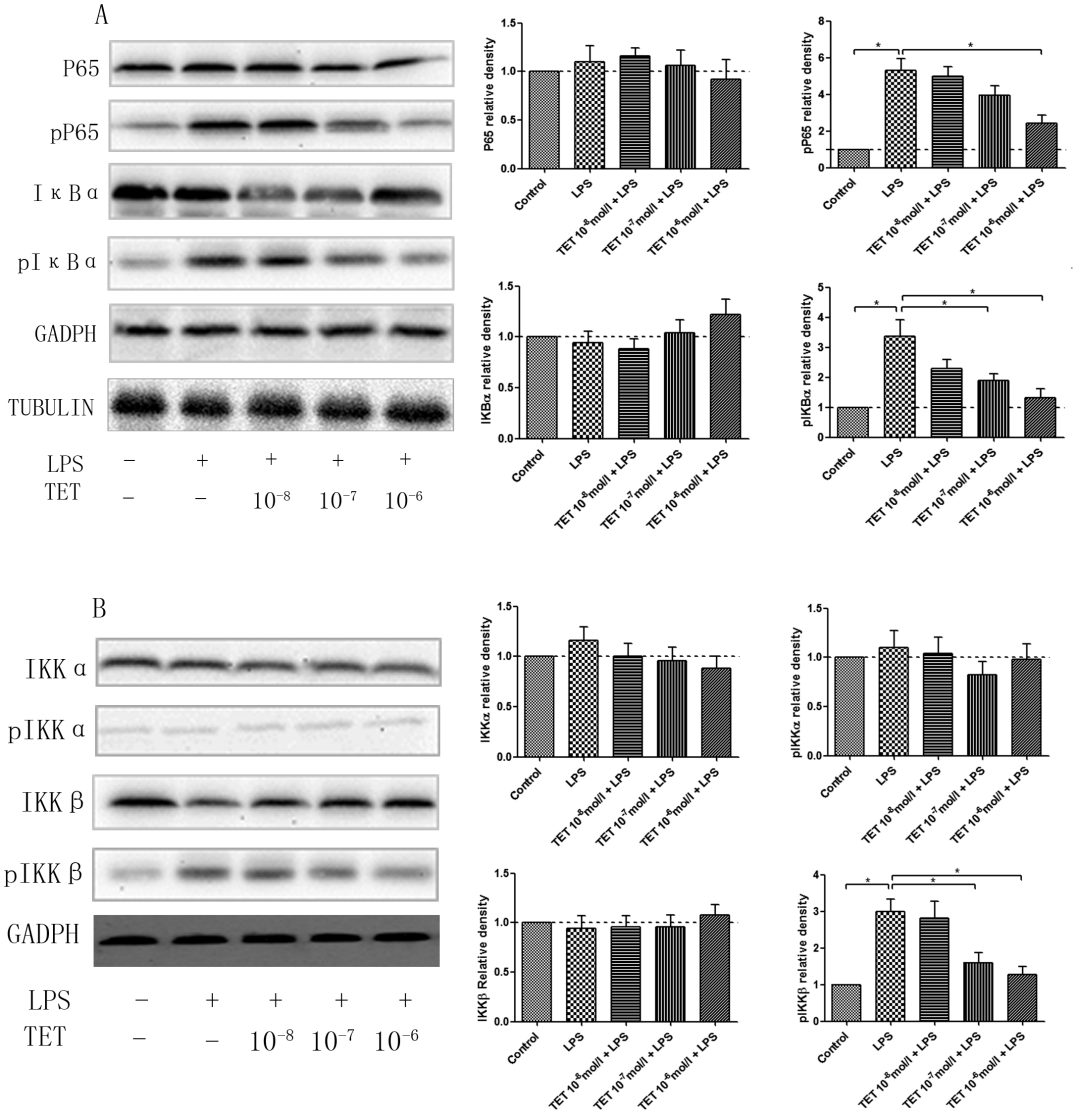
Regulatory effects of TET on the NF-κB pathway in LPS-induced cultured astroglia. (A) The expression of P65, pP65, IκBα, and pIκBα in the presence or absence of TET and LPS was assessed by western blotting, and the quantified comparisons of relative densities are shown. (B) The protein expression of IKKs in the presence or absence of TET and LPS was assessed by western blotting, and the quantified relative densities. Values are shown as M±SD, and normalized to the NS groups. *, *P*<0.05.

The NF-κB signaling pathway is consensually involved in LPS-induced cell activation and inflammation[26], [27]. To investigate whether NF-κB activity is also regulated by TET, the expression of various components of the NF-κB pathway, including P65, pP65, IκBα, and pIκBα, were assessed by western blotting in cultured astroglia. As shown in Figure 4A, P65 expression is not significantly changed in the presence of LPS or TET. However, pP65 was notably up-regulated after LPS stimulus and gradually reversed by TET pretreatment. Meanwhile, pIκBα levels increased dramatically after LPS treatment and decreased with TET pretreatment. No changes in IκBα levels were observed. These results suggest that TET can inactivate the NF-κB signaling pathway through the inhibition of LPS-induced increases in IκBα phosphorylation, thus preventing the degradation of IκBa and retaining NF-κB in the cytoplasm(Fig. 4A).

The phosphorylation of IκBα is catalyzed by the IKK complex, which is comprised of the IKKα, IKKβ, and IKKγ subunits. Among these, IKKα and IKKβ serve as the catalytic subunits to phosphorylate IκB for degradation via ubiquitination[28]. Hence, we investigated whether IKKα and IKKβ are the upstream targets for TET in the NF-κB pathway. Western blotting showed that LPS increased the phosphorylation of IKKβ without affecting IKKα, which was consistent with previous reports[29], [30], [31]. Similarly, TET pretreatment effectively inhibited IKKβ phosphorylation without affecting IKKα (Fig. 4B), which suggests that TET represses IκBα activity by inhibiting IKKβ.

**Figure 5:**
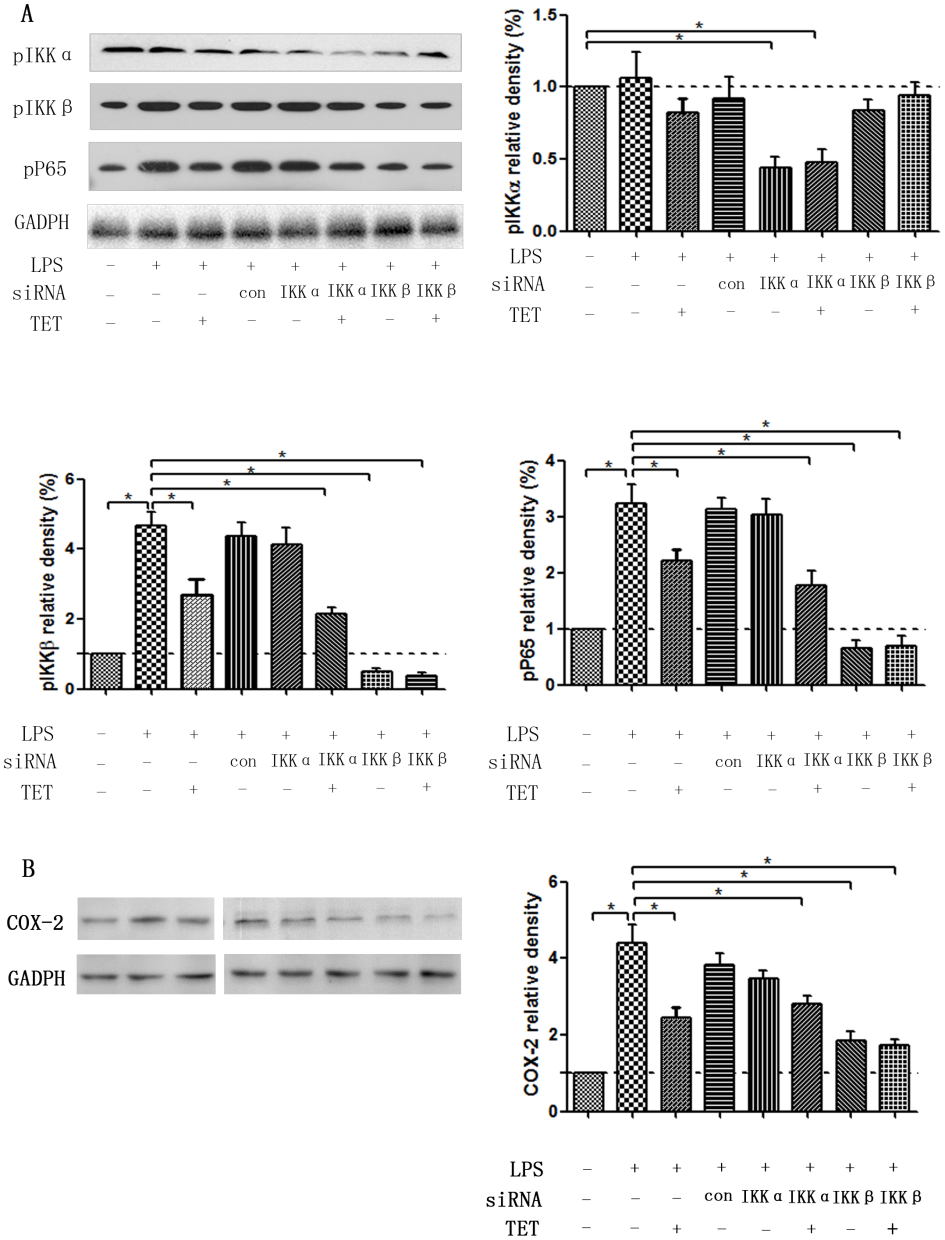
Signaling targets of LPS and TET. Small RNA-interfering (si) experiments with siIKKα, siIKKβ, and control siRNA were performed to investigate the specific targets of LPS and TET in astroglia. (A) Both LPS and TET initially target the phosphorylation of IKKβ, but not that of IKKα. (B) Both siIKKβ and TET decrease COX-2 generation, but not siIKKα. Values are shown as M±SD, and normalized to the LPS(-)siRNA(-)TET(-) groups. *, *P*<0.05.

To verify whether TET specifically targets IKKβ, cells were transfected with IKKα or IKKβ siRNA (si) to knock down their respective gene expression levels. As shown in Figure 5A, cells transfected with siIKKβ exhibit decreases in the protein expression of phosphorylated IKKβ, and pP65. LPS stimulus partially rescued these trends, although not to levels of those seen in LPS-treated control siRNA-transfected cells. SiIKKβ transfection also did not affect IKKα phosphorylation, and phosphorylated IKKβ did not functionally affect pIKKα in the case of LPS challenge, as shown by other groups[32], [33], [34]. On the other hand, transfection of cells with siIKKα only decreased the expression of pIKKα without affecting the levels of pIKKβ or pP65. Collectively, these results show that IKKα and IKKβ played distinct roles in the pathway of LPS-induced hyperalgesia. Moreover, IKKα may not directly participate in the LPS- and TET-signaling cascade. TET treatment in cells with IKKβ knockdown had no effect on the levels of pIKKβ and pP65. This was partially due to the low levels of IKKβ that already existed in these cells with IKKβ knockdown.

Next, to further determine whether the pathway of “LPS/TET—IKKβ—NF-κB—COX-2/PGE_2_” practically take effect in astroglia, we investigated the COX-2 generation under the control siRNA, siIKKα, siIKKβ knockdown with or without TET treatment(Fig 5B). Results showed both siIKKβ and TET could decrease COX-2 generation, but not siIKKα, which more solidly supported the conclusion of TET specifically inhibited IKKβ phosphorylation and subsequently downregulate COX-2/PGE_2_ levels.

## Discussion

LPS, which is a component of the cell wall of gram-negative bacteria, is known to activate a number of cellular signals in various cell types and tissues during inflammation and infection. In addition to its ability to cause endotoxic shock, LPS induces hyperalgesia in mice at lower doses[35]. A single dose of LPS that is administered centrally or peripherally can evoke a hyperalgesic reaction by decreasing mechanical nociceptive thresholds. In this study, we generated a hyperalgesic mouse model, in which BALB/C mice were treated with LPS. The hyperalgesic effect of LPS was verified by the shortened latency time of paw licking using the canonical hot-plate test, as well as the increase in writhing numbers in the acetic acid-induced abdominal constriction test. Because the writhing model is sensitive to the antinociceptive action of non-steroidal anti-inflammatory drugs, including indomethacin[23], and the hot-plate test focuses on the pathophysiological process above the spinal cord level[24], we chose indomethacin and morphine to be the positive controls of the writhing and hot-plate tests, respectively. Using these models, tests, and drugs, we were able to investigate the peripheral and central anti-nociceptive effects of TET.

TET dose-dependently reduced the nociceptive responses in the writhing and hot-plate tests in LPS-treated mice, thus suggesting that TET has both central and peripheral anti-nociceptive effects. Because PGE_2_ is a critical pro-inflammatory and algesic factor, we measured its levels *in vivo* and *in vitro*. PGE_2_ levels were significantly increased and repressed with LPS and TET treatments, respectively, in mouse sera, brain tissues, and cultured astroglia. This suggests that PGE_2_ plays pivotal roles in LPS-induced hyperalgesia and TET-mediated analgesia. The COXs are key enzymes that regulate the formation of PGE_2_ from arachidonic acid. LPS increased COX-2 expression in mouse brain tissues and cultured astroglia. No effects on COX-1 were seen. Consistent with the physiology of canonical pain, COX-2 acted as a key regulatory synthase in the production of PGE_2_ in our hyperalgesic mice and astroglia models. These results show that PGE_2_/COX-2 was the appropriate central pathway of hyperalgesia. Proportional decreases in central and peripheral PGE_2_/COX-2 levels by TET were also observed.

A crucial role for astroglia in mediating pain has been implicated by studies involving animal models and patients with persistent pain conditions[36]. Pro-inflammatory cytokines are produced and released by activated microglia and astrocytes in the CNS. The IKK/IκB/NF-κB signaling pathway regulates the expression of these inflammatory cytokines, including COX-2 and IL-1[37]. Therefore, we isolated astrocytes from the brains of newborn mice and co-treated them with TET and LPS. The phosphorylation of IKKβ, IκBα, P65 and COX-2 increased proportionally upon LPS stimulus, and these increases were significantly reversed by TET co-treatment, thus implicating the IKKβ/IκBα/NF-κB pathway in LPS-induced hyperalgesia and TET-induced antinociception. No effects on IKKα were observed. Knockdown experiments with IKKα or IKKβ siRNAs further clarified the mechanism by which TET elicits its analgesic effects, and the results show that LPS induced NF-κB pathway activation by, at least in part, triggering the phosphorylation of IKKβ but not IKKα. Interestingly, TET specifically targeted IKKβ phosphorylation in LPS-treated astroglia, and eventually depressed NF-Kβ activation and COX-2/PGE_2_ expression. These results allow us to better understand the mechanisms by which LPS and TET induce hyperalgesia and antinociception, respectively, and show that both effects were elicited via the activation or inhibition of IKKβ phosphorylation and the downregulation of the NF-κB/COX-2/PGE_2_ pathway.

Although TET appears to mediate analgesia via inhibiting IKKβ phosphorylation, it may also target other components of the pathway that are upstream of IKK. Additionally, the modulation of pain by peripherally derived inflammatory mediators involves factors and effector cells other than PGE_2_ and astroglia, respectively. The microglia and spinal glia also participate in pain modulation[38], [39]. Whether the central modulation of pain involves the actions of the other eicosanoid metabolites, nitric oxide, or pro-inflammatory mediators requires further elucidation. Therefore, more work needs to be done to reveal the exact mechanisms of hyperalgesia, as well as the main mechanisms behind the analgesic effects of TET.

## References

[1] Ndengele MM, Cuzzocrea S, Esposito E, Mazzon E, Di Paola R, et al (2008) Cyclooxygenases 1 and 2 contribute to peroxynitrite-mediated inflammatorypain hypersensitivity. FASEB J 22(9) 3154–3164. 18497304 10.1096/fj.08-108159

[2] Benson S, Kattoor J, Wegner A, Hammes F, Reidick D, et al (2012) Acute experimental endotoxemia induces visceral hypersensitivity and altered pain evaluation in healthy humans. Pain. 153(4): 794–799. 10.1016/j.pain.2011.12.001 22264996

[3] Xiang Y, Wei X, Chen L, Liu H, Liu X, et al.. (2013) Anti-inflammatory Effect of Acetylpuerarin on Eicosanoid Signaling Pathway in Primary Rat Astrocytes. J Mol Neurosci. Sep 13. (PMID: 24026619) 10.1007/s12031-013-0113-6 24026619

[4] Johann SL, Kampmann E, Denecke B, Arnold S, Kipp M, et al (2008) Expression of enzymes involved in the prostanoid metabolism by cortical astrocytes after LPS-induced inflammation. J Mol Neurosci 34 2: 177–185. 18172772 10.1007/s12031-007-9028-4

[5] Cao L, Tanga FY, Deleo JA (2009) The contributing role of CD14 in toll-like receptor 4 dependent neuropathic pain. Neuroscience. 158(2): 896–903. 10.1016/j.neuroscience.2008.10.004 PMC2701510 18976692

[6] Ma W1, Quirion R (2008) Does COX2-dependent PGE2 play a role in neuropathic pain? Neurosci Lett 437(3): 165–169. 18434017 10.1016/j.neulet.2008.02.072

[7] Southall MD, Vasko MR (2001) Prostaglandin receptor subtypes, EP3C and EP4, mediate the prostaglandin E2-induced cAMP production and sensitization of sensory neurons. J Biol Chem 276(19): 16083–16091. 11278900 10.1074/jbc.M011408200

[8] Berenbaum F (2004) COX-3: fact or fancy? Joint Bone Spine 71: 451–453. 15589421 10.1016/j.jbspin.2004.02.003

[9] Nasrallah R, Clark J, Hébert RL (2007) Prostaglandins in the kidney:developments since Y2K. Clin Sci (Lond) 113(7): 297–311. 17760567 10.1042/CS20070089

[10] Kanngiesser M, Häussler A, Myrczek T, Küsener N, Lim HY, et al (2012) Inhibitor kappa B kinase beta dependent cytokine upregulation in nociceptive neurons contributes to nociceptive hypersensitivity after sciatic nerve injury. J Pain 13(5): 485–497. 22564672 10.1016/j.jpain.2012.02.010

[11] Hsu CC, Lien JC, Chang CW, Chang CH, Kuo SC, et al (2013) Yuwen02f1 suppresses LPS-induced endotoxemia and adjuvant-induced arthritis primarily through blockade of ROS formation, NFkB and MAPK activation. Biochem Pharmacol 85(3): 385–395. 23142712 10.1016/j.bcp.2012.11.002

[12] Haddad JJ, Abdel-Karim NE (2011) NF-κB cellular and molecular regulatory mechanisms and pathways: therapeutic pattern or pseudoregulation? Cell Immunol 271(1): 5–14. 21777910 10.1016/j.cellimm.2011.06.021

[13] Niederberger E, Geisslinger G (2008) The IKK-NF-kappaB pathway: a source for novel molecular drug targets in pain therapy? FASEB J 22(10): 3432–3442. 18559989 10.1096/fj.08-109355

[14] Yu XC, Wu S, Chen CF, Pang KT, Wong TM, et al (2004) Antihypertensive and anti-arrhythmic effects of an extract of Radix Stephaniae Tetrandrae in the rat. J Pharm Pharmacol 56(1): 115–122. 14980008 10.1211/0022357022458

[15] Iturriaga-Vásquez P, Miquel R, Ivorra MD, D'Ocon MP, Cassels BK, et al (2003) Simplified tetrandrine congeners as possible antihypertensive agents with a dual mechanism of action. J Nat Prod 66(7): 954–957. 12880313 10.1021/np030022+

[16] Shen DF, Tang QZ, Yan L, Zhang Y, Zhu LH, et al (2010) Tetrandrine blocks cardiac hypertrophy by disrupting reactive oxygen species-dependent ERK1/2 signalling. Br J Pharmacol 159(4): 970–981. 20105174 10.1111/j.1476-5381.2009.00605.x PMC2829222

[17] Li X, Su B, Liu R, Zhang Y, Zhu LH, et al (2011) Tetrandrine induces apoptosis and triggers caspase cascade in human bladder cancer cells. J Surg Res 166(1): e45–51. 21176918 10.1016/j.jss.2010.10.034

[18] Lee YS, Han SH, Lee SH, Kim YG, Park CB, et al (2012) The mechanism of antibacterial activity of retrandrine against Staphylococcus arreus. Foodborne Pathog Dis 9(8): 686–691. 22845553 10.1089/fpd.2011.1119

[19] Gong X, Luo FL, Zhang L, Li HZ, Wu MJ, et al (2010) Tetrandrine attenuates lipopolysaccharide-induced fulminant hepatic failure in D-galactosamine-sensitized mice. Int Immunopharmacol 10(3): 357–363. 20036342 10.1016/j.intimp.2009.12.010

[20] Wang TH, Wan JY, Gong X, Li HZ, Cheng Y (2012) Tetrandrine enhances cytotoxicity of cisplatin in human drug-resistant esophageal squamous carcinoma cells by inhibition of multidrug resistance-associated protein 1. Oncol Rep 28(5): 1681–1686. 22941407 10.3892/or.2012.1999

[21] Miyachi T, Asai K, Tsuiki H, Mizuno H, Yamamoto N, et al (2001) Interleukin-1β induces the expression of lipocortin 1 mRNA in cultured rat cortical astrocytes. Neurosci. Res 40(1): 53–60. 11311405 10.1016/s0168-0102(01)00208-5

[22] Thomsen R, Daugaard TF, Holm IE, Nielsen AL (2013) Alternative mRNA Splicing from the Glial Fibrillary Acidic Protein (GFAP) Gene Generates Isoforms with Distinct Subcellular mRNA Localization Patterns in Astrocytes. PLoS One 8(8): e72110. 23991052 10.1371/journal.pone.0072110 PMC3753360

[23] Kaushik D, Kumar D, Kaushik P, Rana AC (2012) Analgesic and Anti-Inflammatory Activity of Pinus roxburghii Sarg. Adv Pharmacol Sci 2012: 245431. 22761611 10.1155/2012/245431 PMC3384912

[24] Ballou LR, Botting RM, Goorha S, Zhang J, Vane JR (2000) Nociception in cyclooxygenase isozyme-deficient mice. Proc Natl Acad Sci USA 97(18): 10272–10276. 10954756 10.1073/pnas.180319297 PMC27868

[25] Morham SG, Langenbach R, Loftin CD, Tiano HF, Vouloumanos N, et al (1995) Prostaglandin synthase 2 gene disruption causes severe renal pathology in the mouse. Cell 83(3): 473–482. 8521477 10.1016/0092-8674(95)90125-6

[26] Koppula S, Kim WJ, Jiang J, Shim DW, Oh NH, et al (2013) Carpesium macrocephalum attenuates lipopolysaccharide-induced inflammation in macrophages by regulating the NF-κB/IκκB-α, Akt, and STAT signaling pathways. Am J Chin Med 41(4): 927–943. 23895161 10.1142/S0192415X13500626

[27] Gao F, Ding B, Zhou L, Gao X, Guo H, et al (2013) Magnesium sulfate provides neuroprotection in lipopolysaccharide-activated primary microglia by inhibiting NF-κB pathway. J Surg Res 184(2): 944–950. 23628437 10.1016/j.jss.2013.03.034

[28] Hayden MS, Ghosh S (2008) Shared principles in NF-kappaB signaling. Cell 132(3): 344–362. 18267068 10.1016/j.cell.2008.01.020

[29] Yang F, Tang E, Guan K, Wang CY (2003) IKK beta plays an essential role in the phosphorylation of RelA/P65 on serine 536 induced by lipopolysaccharide. J Immunol 170(11): 5630–5635. 12759443 10.4049/jimmunol.170.11.5630

[30] Dajani R, Sanlioglu S, Zhang Y, Li Q, Monick MM, et al (2007) Pleiotropic functions of TNF- alpha determine distinct IKKbeta-dependent hepatocellular fates in response to LPS. Am J Physiol Gastrointest Liver Physiol 292(1): G242–252. 16935850 10.1152/ajpgi.00043.2006

[31] Shao DZ, Lin M (2008) Platonin inhibits LPS-induced NF-kappaB by preventing activation of Akt and IKKbeta in human PBMC. Inflamm Res 57(12): 601–606. 19109741 10.1007/s00011-008-8053-2

[32] Li ZW, Chu W, Hu Y, Delhase M, Deerinck T, et al (1999) The IKKbeta subunit of IkappaB kinase (IKK) is essential for nuclear factor kappaBactivation and prevention of apoptosis. J Exp Med 189(11): 1839–1845. 10359587 10.1084/jem.189.11.1839 PMC2193082

[33] Park EJ, Cheenpracha S, Chang LC, Pezzuto JM (2011) Suppression of cyclooxygenase-2 and inducible nitric oxide synthase expression by epimuqubilin A via IKK/IκB/NF-κB pathways in lipopolysaccharide-stimulated RAW 264. 7 cells. Phytochem Lett 4(4): 426–431. 22180763 10.1016/j.phytol.2011.07.009 PMC3237698

[34] Islam S, Hassan F, Mu MM, Ito H, Koide N, et al (2004) Piceatannol prevents lipopolysaccharide (LPS)-induced nitric oxide (NO) production and nuclear factor (NF)-kappaB activation by inhibiting IkappaB kinase (IKK). Microbiol Immunol 48(10): 729–736. 15502405 10.1111/j.1348-0421.2004.tb03598.x

[35] Kovács KJ, Papic JC, Larson AA (2008) Movement-evoked hyperalgesia induced by lipopolysaccharides is not suppressed by glucocorticoids. Pain 136(1–2): 75–84. 17686584 10.1016/j.pain.2007.06.017 PMC2430893

[36] Svensson CI, Brodin E (2010) Spinal astrocytes in pain processing: non-neuronal cells as therapeutic targets. Mol Interv 10(1): 25–38. 20124561 10.1124/mi.10.1.6

[37] Shishodia S, Koul D, Aggarwal BB (2004) Cyclooxygenase (COX)-2 inhibitor celecoxib abrogates TNF-induced NF-kappa B activation through inhibition of activation of I kappa B alpha kinase and Akt in human non-small cell lung carcinoma: correlation with suppression of COX-2 synthesis. J Immunol 173(3): 2011–2022. 15265936 10.4049/jimmunol.173.3.2011

[38] Saito O, Svensson CI, Buczynski MW, Wegner K, Hua XY, et al (2010) Spinal glial TLR4-mediated nociception and production of prostaglandin E(2) and TNF. Br J Pharmacol 160(7): 1754–1764. 20649577 10.1111/j.1476-5381.2010.00811.x PMC2936846

[39] Ikeda-Matsuo Y, Ikegaya Y, Matsuki N, Uematsu S, Akira S, et al (2005) Microglia-specific expression of microsomal prostaglandin E2 synthase-1 contributes to lipopolysaccharide-induced prostaglandin E2 production. J Neurochem 94(6): 1546–1558. 16000148 10.1111/j.1471-4159.2005.03302.x

